# Editorial: Plant specialized metabolism for plant protection: Genomics and biotechnology

**DOI:** 10.3389/fpls.2022.1080939

**Published:** 2022-11-15

**Authors:** Zhihua Liao, Lei Zhang, Felipe Vázquez-Flota

**Affiliations:** ^1^ State Key Laboratory of Silkworm Genome Biology, Integrative Science Center of Germplasm Creation in Western China (CHONGQING) Science City & Southwest University, SWU-TAAHC Medicinal Plant Joint R&D Centre, School of Life Sciences, Southwest University, Chongqing, China; ^2^ Chongqing Academy of Science and Technology, Chongqing, China; ^3^ Department of Pharmaceutical Botany, School of Pharmacy, Naval Medical University, Shanghai, China; ^4^ Unidad de Bioquímica y Biología Molecular de Plantas, Centro de Investigación Científica de Yucatán, Mérida, Yucatán, México

**Keywords:** biosynthesis, chemical diversity, plant-environment interactions, specialized metabolites, transcriptional factors

## Introduction

The philosophical conception of the now called plant specialized metabolites (PSM) has radically changed over the last three decades. Up to the late 1980’s, it was common to find extensive literature reviews analyzing conditions leading to the synthesis of these low molecular weight compounds and discussing their probable physiological roles. Despite of numerous reports of chemically mediated plant-insect interactions already available at the time, they were considered as metabolic waste or a simple means to keep major metabolic pathways in operation (Haslam, 1986; Williams et al., 1989). This apparently superfluous character was the reason to refer them as *secondary metabolites*, a term that persists in the plant science’s vocabulary up to now. Regardless of semantic preferences, all these compounds share some important structural and biological features, such as displaying a wide chemical diversity and presenting a limited taxonomical distribution. Besides, they are accumulated in only tiny amounts and under specific conditions. Most importantly, they exert physiological activities on other organisms.

However, as the molecular mechanism controlling PSM formation in response to environmental cues were discovered, it became evident that these compounds were part of a plant sophisticated set of chemical tools to interact with their surroundings (Erb and Kliebenstein, 2020; Jamil et al., 2020). With this, new venues were opened for these specialized chemicals’ which for a long time, had been profited from straightforward processes of extraction-purification and product elaboration (Salim and De Luca, 2013).

This collection on Plant Specialized Metabolism for Plant Protection: Genomics and Biotechnology, illustrates the quick progress experienced in this field as the high-throughput technologies are turning into common tools for non-model species. Most articles nicely merge metabolomic and transcriptomic data, drawing an integral view of the operating metabolic networks and the possible master genes that coordinate them. Ultimately, two aspects of PSM were taken as main motives for this collection; the transcriptional factors (TF) acting as such master regulatory genes and the environmental conditions that activate the intricate biosynthetic pathway involved.

## Transcriptional control of plant specialized metabolism

Combining anthocyanin profiling and transcriptomic analysis of differentially colored flowers of *Fressia hybrida*, Zhu et al. reported the finding of four novel TFs, from the WRKY and AP2 protein families, involved in governing petal’s colour. A similar approach was followed by Yang et al. to address the basis of colour differences in white and pink *Lycoris sprengeri* flowers.

Moreover, Shen et al. described the coordinated interplay of bHLH and MYC2 proteins for switching on and off artemisinin biosynthesis in *Artemisa annua*. This work not just reveals a novel mechanism for the fine tuning of the biosynthesis of this metabolite, but also opens biotechnological opportunities, considering the medicinal applications of this plant. The associations between growth promotion, specifically trough cell wall formation, and the synthesis of specialized metabolites, are elegantly addressed by Yu et al, who traced down domestication traits in *Panax notoginseng*. A new MYB protein, able to bind to *cis* elements in promotors from genes for cell wall and ginsenoside biosynthesis, was found in this work which also discussed how human selection with medicinal purposes greatly influenced root chemical composition. Genes coding for proteins of the relatively small, and less studied Teosinte branched1/Cycloidea/Proliferating cell factor (TCP) family are proposed as possible modulators of camptothecin formation, by Wang et al., through genomic analysis of *Ophiorriza pumila*. Candidates involved, in both forming these alkaloids, and furnishing precursors to the biosynthetic pathway, were described and analyzed with regard to their cell distribution and ability to activate selected promotors in this medicinally important Asian plant.

Besides these *trans* functioning elements, the role of chromatin conformation was analyzed in *Cannabis sativa* by Yang et al.. Up to 14 putative histone deacetylase genes displayed matching transcriptional profiles to a set of selected cannabinoids biosynthetic genes. A particularly interesting result is that five of them, which belong to the C subfamily were affected along cannabinoid formation in plantlets treated with Trichostatin A, a broad inhibitor of their action. This work is one of the few reports dealing with specialized metabolism at this early regulatory mechanism.

Additionally to these studies, Zhan et al. presented a comprehensive review linking metabolic pathways with the environmental stimuli triggering them and the responsible genetic regulators. A careful choice of examples among alkaloids, glucosinolates, terpenoids, phenols and flavonoids was included by the authors. As for alkaloids, Song et al. covers the state of the art on the distribution of alkaloids from different families and transcriptomic data in *Dendrobium* medicinal species. Based on the combined information, biosynthetic routes are proposed, setting the stage for actual gene isolation and further studies in this interesting plant.

## Specialized metabolism in environmental interactions

The adaptative role of specialized metabolites under adverse plant-environment interactions has been profusely documented. In this issue, Peng et al analyzed two cultivars of *Angelica sinensis* exposed to enhanced UV-B radiation. A contrasting metabolic performance was found, related to ferulic acid and flavonoid synthesis. ROS scavenger systems were also analyzed in this work, and important variations in the ascorbate-glutathione cycle were found. An interesting study of the superoxide dismutase (SOD) gene family in protecting tobacco plants exposed to heavy metal was presented by Huo et al.. Effects of other metals, such as copper, were also approached in *Salvia mithiorrhiza*. Combining the data from metabolomics and transcriptomics, Xiang et al. found an increase in salvianolic acid accumulation that could be associated with a significative increase of laccase transcripts and the occurrence of specific MYB and zinc finger proteins.

On the other hand, since photovoltaic agriculture is quickly spreading as a promising sustainable farming alternative, a better understanding of plant adaptative mechanisms to these conditions is required. The study of Xie et al. on the effects of photovoltaic conditions on performance of the medicinal plant *Tetrastigma hemsleyanum* revealed a decrease in photosynthetic efficiency which could be alleviated by the external addition of stress hormones, such as jasmonate and salicylic acid (SA). An interesting interplay between SA and NO modulating photovoltaic adaptation was described in this work, which also showed that these innovative agricultural practises could be adapted to SM producing plants.

## Plant specialized metabolism. The road ahead

Conservative estimations fix the number of phytochemicals in the order of hundred of thousands. Although many of them result from the occurrence of chiral centers and different modification patterns on a common core structure, such wide diversity involves the operation of elaborate biochemical networks and regulation at the genetic level. Comprehensive ‘omics’ approaches to plant specialized metabolism had unveiled some remarkable differences from primary metabolism, which explain the basis for this impressive phytochemical diversity. Each plant presents its unique chemical mixture, which results from the particular tuning of its genetic potential and developmental program with the surroundings. The increased access of high-throughput technologies has allowed us to expand our knowledge on the critical points for reaching this chemical setting. Moreover, these tools also present opportunities for engineering metabolic and synthetic biology strategies for more sustainable uses of these valuable natural resources.

## Author contributions

FV-F wrote the first draft, ZL and LZ corrected and provided input to the first and subsequent drafts. All authors contributed to the article and approved the submitted version.

## Funding

FV-F received funding from National Research Council for Science and Technology (CONACYT México; CB-2016-0285887). LZ was financially supported by the National Natural Science Foundation of China (31970316;32170274;82225047) and National Key R&D Program of China (2022YFC3500098). ZL received funding from the NSFC project (81973420) and the National Key Research and Development Project (2019YFE0108700).

## Acknowledgments

The authors wish to thank to all the contributors and the FPS editorial team for their efficient work. We would like to express our deep gratitude to all reviewers for their valuable participation in assuring the scientific quality of this collection. FV-F wish to thank Dr. ML Miranda-Ham for critical reviewing the first draft.

## Conflict of interest

The authors declare that the research was conducted in the absence of any commercial or financial relationships that could be construed as a potential conflict of interest.

## Publisher’s note

All claims expressed in this article are solely those of the authors and do not necessarily represent those of their affiliated organizations, or those of the publisher, the editors and the reviewers. Any product that may be evaluated in this article, or claim that may be made by its manufacturer, is not guaranteed or endorsed by the publisher.
